# Influence of Accelerated Carbonation Conditions on the Physical Properties Improvement of Recycled Coarse Aggregate

**DOI:** 10.3390/ma18040901

**Published:** 2025-02-19

**Authors:** Nasir Mehmood, Pinghua Zhu, Hui Liu, Haichao Li, Xudong Zhu

**Affiliations:** School of Urban Construction, Changzhou University, Changzhou 213164, China; jannasir965@gmail.com (N.M.); liuhui@cczu.edu.cn (H.L.); cczulihaichao@163.com (H.L.); jsyczxd222@gmail.com (X.Z.)

**Keywords:** carbon dioxide curing, recycled coarse aggregate, relative humidity, accelerated carbonation, CO_2_ curing

## Abstract

The preparation of new-generation concrete from recycled coarse aggregate (RA) is an effective way to realize the resource utilization of construction waste. However, loose and porous attached mortar leads to RA showing low-density, high-water absorption, and high crushing value. However, carbonation modification treatment can effectively improve the performance of RA. This paper studied the effects of carbon dioxide (CO_2_) concentration, gas pressure, and moisture content on the RA physical properties (apparent density, water absorption, crushing value, and soundness) of waste concrete. The results showed that, when the (CO_2_) concentration increased from 20% to 60%, the apparent density of RA after carbonation increased by 0.23–0.31%, the water absorption decreased by 0.57–0.93%, the crushing value decreased by 0.36–0.61%, and the soundness decreased by 0.47–0.85%. When the (CO_2_) concentration was further increased from 60% to 80%, the apparent density of RA after carbonation was increased by 0.04–0.05%, the water absorption was improved by 0.15–0.31%, the crushing value was reduced by 0.06–0.07%, and the soundness was reduced by 0.09–0.11%. During the carbonation modification process, the performance of RA was significantly enhanced when the moisture content was 3.4% and the dissolution of hydration products was accelerated. The diffusion rate of CO_2_ and the carbonation reaction rate decreased with the high moisture content of RA. As gas pressure increases to 0.01 MPa, the physical properties of RA change significantly, because gas pressure promotes the carbonation reaction between hydration products and CO_2_ in attached mortar. As the gas pressure increased to 0.5 MPa, RA’s apparent density gradually increased, while its water absorption, crushing value, and stability gradually decreased. The result improved RA’s performance. SEM images show that carbonation modification of RA under different gas pressures increases CaCO_3_ in attached mortar, filling the Interfacial Transition Zone (ITZ), and decreasing crack width and number. Gas pressure accelerates CO_2_ diffusion and reaction with hydration products, resulting in narrower ITZ and dense mortar.

## 1. Introduction

Because of the rapid increase in urbanization and infrastructure construction, a wider range of natural resources is becoming scarce [1,2]. Concurrently, the quantity of construction and demolition waste generated from buildings is increasing, representing a significant environmental hazard due to its occupation of valuable land [3,4]. In structural concrete, recycled coarse aggregate (RA) from crushed waste concrete is a common substitute for natural coarse aggregate. This approach helps address the challenges of natural resource scarcity and construction waste management [5,6]. There is a notable economic and environmental benefit to be gained from utilizing waste concrete as RA [7]. Kępniak et al. [8] conducted a carbon footprint analysis of the process of recycling waste concrete and found that the use of recycled aggregates can significantly reduce the consumption of energy and raw materials, offering clear environmental and economic benefits. However, the durability and mechanical properties of concrete often proved inadequate when RA was employed, as demonstrated by Xiao et al. [9,10]. Researchers have found that applying RA considerably decreased the resistance to chloride ion permeability when compared to natural concrete. Furthermore, the application of RA also demonstrated a reduction in the freezing resistance of recycled concrete [11,12]. These issues were caused by the inferior Interfacial Transition Zones between the new cement mortar and the aggregate in RA, which prevented the old mortar from adhering effectively [13,14]. When compared to natural coarse aggregate, RA has higher water absorption, lower apparent density, higher crushing value, and more porosity [15,16]. The modification of recycled aggregate is a matter of great urgency in the engineering application of recycled concrete [17].

Recently, the prevailing methodologies for RA modification have been predominantly categorized into two principal approaches: removal and reinforcement [18,19]. The removal procedure entails employing technology to remove the connected mortar from the surface of the RA, such as grinding, acid washing, or heating. The principal reinforcement techniques for attached mortar are based on carbonation, and polymer treatments [20,21]. To increase the qualities of the RA, a process that is reasonable, eco-friendly, as well time-efficient must be developed [22]. The adoption of most reinforcement techniques has a negative influence on the environment, while also representing a significant cost [23,24,25]. In comparison, carbonation is the most cost-effective and environmentally beneficial modification process [26,27,28].

The carbonation reaction allows cement-based materials to form dense silica gels and CaCO_3_, which can densify the existing mortar attached to RA and the pores in the old Interfacial Transition Zone (ITZ) [29,30]. In comparison to the untreated RA, the carbonation process has been shown to enhance the physical properties of the RA, improving its apparent density and reducing its water absorption and crushing value [31,32]. This process strengthens the microstructure of the material and improves the macroscopic properties of RA [33,34]. Furthermore, it reduces the drying loss of volume, water absorption, and the chloride migration coefficient of concrete [35,36,37]. Mortars made with carbonated recycled fine aggregate also exhibited better flow and consistency loss values, according to a detailed examination of the effect of carbonation modification on recycled fine aggregate concrete performance [38,39].

Carbonation modification has been proven to be an effective and environmentally friendly method for enhancing the performance of recycled aggregates. Farahani et al. [40] compared the costs associated with using carbonated RA and natural aggregates (NAs). The cost of using 1 ton of RA was found to be $15, while the cost of NA was $25, resulting in a savings of approximately $10 per ton. It is estimated that implementing the 50% substitution rate is estimated to help save $9000 million each year in the United States, which signifies an economic benefit for the sustainable development of the construction industry. Zhang et al. [41] also found that, in the United States, every ton of carbonized recycled aggregate can save $ 18.5. Zhang et al. also found that 1-ton carbonated RA can result in a savings of $18.5 in the United States. However, specific and optimal carbonation conditions, such as temperature, relative humidity, carbonation pressure, and CO_2_ concentration, have not been fully explored. These factors significantly influence both the effectiveness of the modification and its associated costs [28]. For instance, high-pressure environments can enhance the dissolution rate of carbon dioxide, thereby accelerating the reaction kinetics and improving the carbonation effect. However, maintaining high-pressure environments requires specialized high-pressure equipment, such as high-pressure reactors, which involve substantial investment and operational maintenance costs. On the other hand, low-pressure carbonation, while reducing the costs of equipment and energy, results in lower reaction efficiency, potentially requiring longer treatment durations, thus increasing unit costs. Our previous research showed that the optimal temperature and relative humidity were 50 °C and 55%, respectively. Given that the carbonation pressure and CO_2_ concentration in the modification process are critical issues for cost control, the exact parameters must be identified [42,43].

In conclusion, carbonation modification has been extensively studied as a method to enhance the performance of recycled aggregates. However, research on the effects of carbonation pressure, CO_2_ concentration, and the moisture content of recycled aggregates on carbonation efficiency remains scarce. The objective of this study was to assess the influence of CO_2_ concentration and gas pressure on the carbonation modification in the physical properties of RA. The physical properties include apparent density, water absorption, crushing value, and soundness. Furthermore, the optimal moisture content in RA was determined. The conclusion can improve the influence of carbonation conditions on the modification efficiency and obtain the optimal values of various carbonation conditions, which can provide a reference for the setting of carbonation parameters, especially in mass production.

## 2. Materials and Methods

### 2.1. Materials

**Figure 1:**
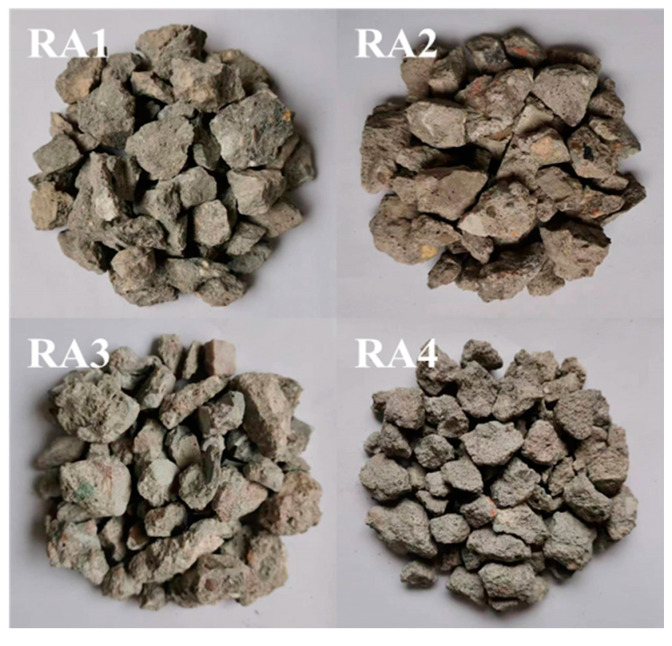
Appearance of RA.

**Figure 2:**
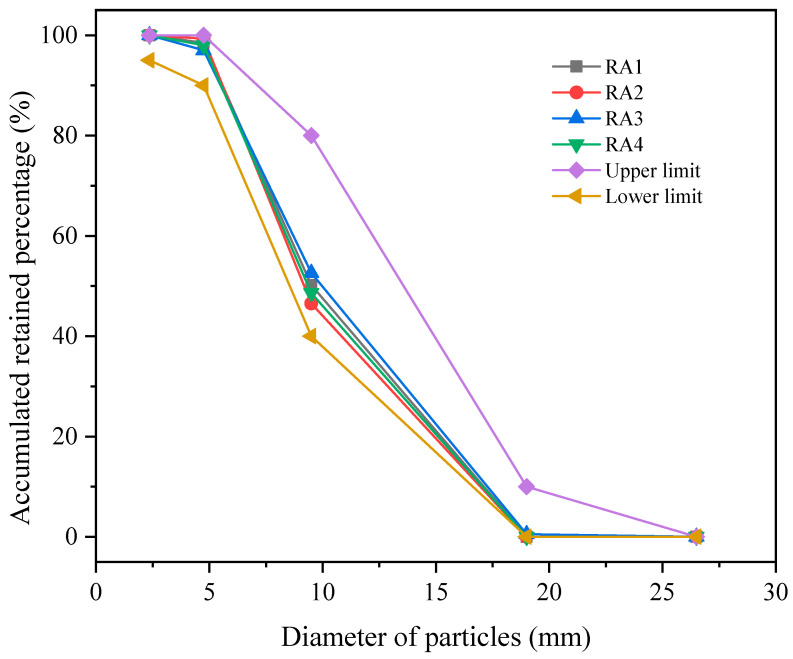
Gradation curves of RA.

Four different types of RA, ranging in size from 5 to 20 mm as shown in Figure 1, were supplied by Jiangsu Lvhe Environmental Technology Co Ltd. The gradation curves of RA are shown in Figure 2. According to Chinese standard GB/T 25177-2010 [44], all the RCAs used in this experiment meet the requirements of continuous gradation. The physical properties of RA are listed in Table 1. The test sample should have a minimum mass of 8 kg when evaluating the apparent density and water absorption rate. For the test for crushing value and firmness, the sample mass should not be less than 3 kg and 1.5 kg, respectively. Each test should be conducted at least three times, and the arithmetic mean value is taken as the final recycled aggregate performance.

### 2.2. Methodology

#### Carbonation Modification

**Figure 3:**
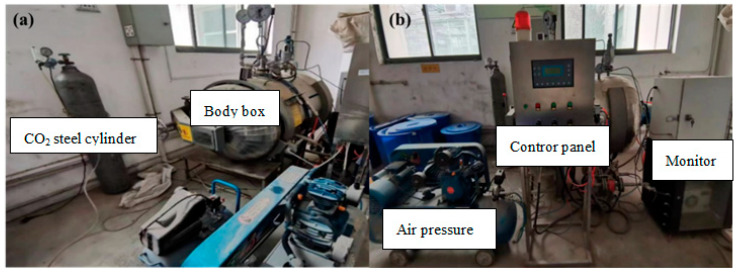
Carbonation modification chamber: (**a**) side face; (**b**) front face.

A pressurized sealed steel kettle with a volume of 400 L is used as the carbonation modification box, as shown in Figure 3. The RA carbonation modification test procedures are as follows. First, place the four types of RA flatly on the tray, put them into the carbonation modification box, and then close the box door. Open the control panel and set the carbonation modification parameters (duration, relative humidity, temperature, CO_2_ concentration, and gas pressure). In the carbonation modification test, industrial grade CO_2_ with a purity higher than 99.9% was used for RA carbonation modification. The heating rate of the carbonation modification box is 10 °C/min. The carbonation modification box is connected to a water tank to increase humidity inside the box. The fan on the right side of the box is used for heat dissipation and dehumidification. The gas pressure in the carbonation modification box is adjusted by an air pump.

The specific parameters of the carbonation modification, including CO_2_ concentration (20%, 40%, 60%, and 80%) and gas pressure (0, 0.01, 0.5, and 1.0 MPa). In addition, different moisture content (3.2%, 3.4%, 3.6%, and 3.8%) of RA was employed. The carbonation temperature and the relative humidity were set as 20 °C and 60%, respectively. Previous research has pointed out that the carbonation period was 48 h, which was revealed to be an ideal duration of time [45] (Table 2).

### 2.3. Measurement

Before and after the carbonation modification, the physical properties of RCAs including apparent density, water absorption, crushing value, and soundness were evaluated. These tests were carried out in strict accordance with GB/T 14685-2022 [46]. After RA carbonation, it was prepared into blocks with a length, width, and height less than 0.5 cm, and then it was tested by SEM after gold spraying. Information regarding the presence and characteristics of elements such as ITZs, pores, and cracks within the RA was acquired through the analysis of SEM images generated for each group using the equipment [45,47].

## 3. Results and Discussion

### 3.1. Effect of CO_2_ Concentration on Physical Properties of RA

**Figure 4:**
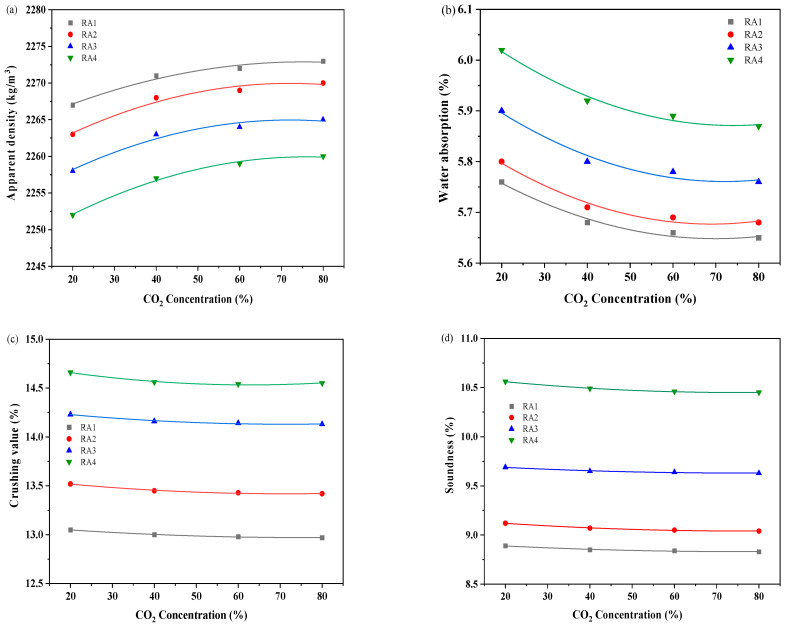
The physical properties of RA after carbonation modification with various CO_2_ concentrations. (**a**)Apparent density; (**b**)water absorption; (**c**) crushing value; (**d**) soundness.

Figure 4 displays the physical characteristics of RA after carbonation modification with different CO_2_ concentrations. As the CO_2_ concentration increases, the apparent density of RA after carbonation modification gradually increases, and the water absorption, crushing value, and solidity gradually decrease. Specifically, in the process of carbonation modification, the improvement of RA physical properties was not significant when CO_2_ concentration exceeded 60%. When the CO_2_ concentration increased from 20% to 60%, the apparent density of RA after carbonation increased by 0.23–0.31%, the water absorption decreased by 0.57–0.93%, and the crushing value decreased by 0.36–0.61%. When the CO_2_ concentration was further increased from 60% to 80%, the apparent density of RA after carbonation was increased by 0.04–0.05%, and the water absorption was improved by 0.15–0.31%. RA physical properties seem not sensitive to high CO_2_ concentration. This is consistent with the research conclusion of Yang et al. [41]. The rate at which CO_2_ diffused into the interior of the RA was linked to CO_2_ concentration and aggregate porosity [48,49]. When the aggregate porosity was certain, the high concentration of CO_2_ had little influence. Furthermore, as CO_2_ concentrations grew, crushing value and soundness decreased, while the changes appeared small. CaCO_3_ generated by carbonation modification had relatively little effect on improving the crushing value and durability of the aggregate.

**Figure 5:**
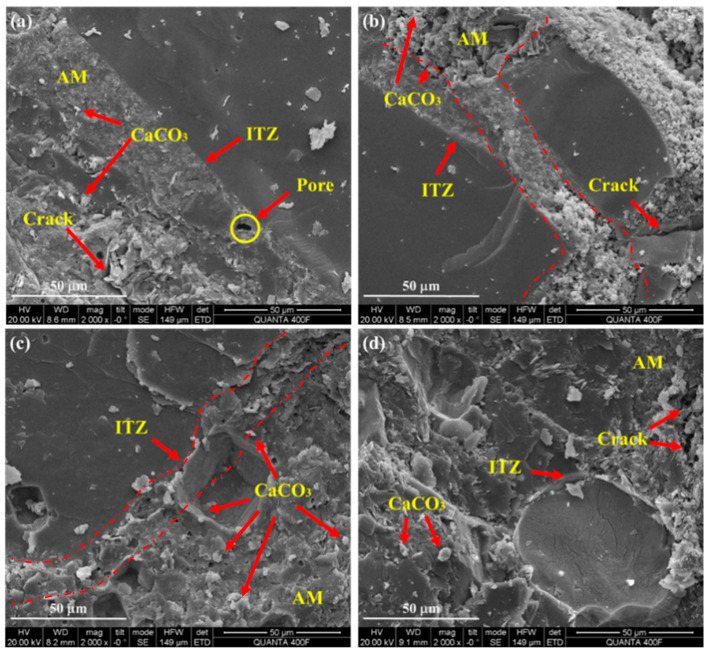
Microscopic SEM images of RA after carbonation modification with various CO_2_ concentrations: (**a**) 20%; (**b**) 40%; (**c**) 60%; (**d**) 80%.

Figure 5 shows the microscopic SEM images of RA after carbonation modification with different CO_2_ concentrations. When the concentration of CO_2_ increases from 20% to 60%, the carbonation product CaCO_3_ gradually increases, filling the pores of ITZ between the attached mortar and the original aggregate. In addition, the crack width in the attached mortar was reduced, which was also the result of carbonation product filling. CO_2_ can enter RA through the pore and react with calcium ions, while the high porosity ITZ is more likely to form calcium carbonate. When CO_2_ concentration further increased to 80%, the attached mortar became regular and no obvious cracks appeared in the attached mortar, which was also the result of carbonation product filling, indicating that the compactness of the attached mortar further increased. In addition, higher CO_2_ concentrations lead to rapid densification of the microstructure of the gelled material, thereby reducing CO_2_ diffusion. At the same time, the carbonation modification rate of RA is also affected by the dissolution rate of hydration products [28,50]. Therefore, the RA performance at 80% CO_2_ concentration is comparable to that at 60% CO_2_ concentration. The microscopic observation provides a basis for the change in RA physical properties after carbonation modification. Therefore, the carbonation modification rate of RA will not increase significantly if the concentration of CO_2_ is too high.

### 3.2. Effect of Carbonation Pressure on the Physical Properties of RA

**Figure 6:**
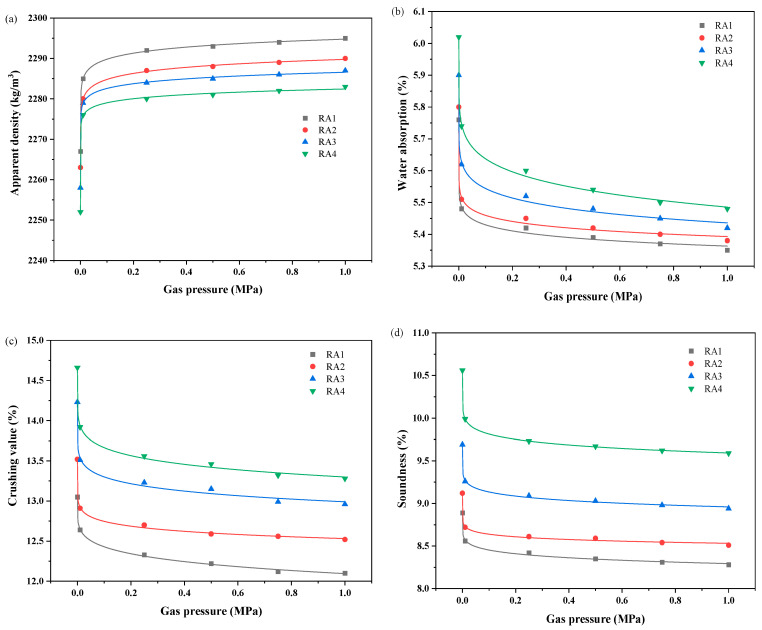
The variation physical properties of RA after carbonation modification under various gas pressures: (**a**) apparent density; (**b**) water absorption (**c**); crushing value; (**d**) soundness.

Figure 6 depicts the effect of carbonation gas pressure on the physical properties of RA. According to the change in gas pressure and the rate of change in the physical properties of RA, the carbonation modification of RA can be divided into three stages: the rapid modification stage (0–0.01 MPa), the slow modification stage (0.01–0.5 MPa), and the stable modification stage (0.5–1.0 MPa). Shuvo et al. [51] and Pu et al. [52] have also obtained similar trend curves. As the carbonation modification gas pressure increases to 0.01 MPa, the physical properties of RA change significantly. This is because the gas pressure promotes the hydration product undergoing a carbonation reaction with CO_2_. As the carbonation modification gas pressure increases to 0.5 MPa, the apparent density of RA gradually increases, and the water absorption, crushing value, as well as solidity gradually decrease. However, when the gas pressure exceeded 0.5 MPa, the performance of RA was improved but not significantly. Therefore, too high gas pressure cannot significantly improve the degree of RA carbonation [53,54]. In general, from the perspective of cost-effectiveness and carbonization efficiency, the CO_2_ concentration of 0.01 MPa is the preferred carbonation condition.

**Figure 7:**
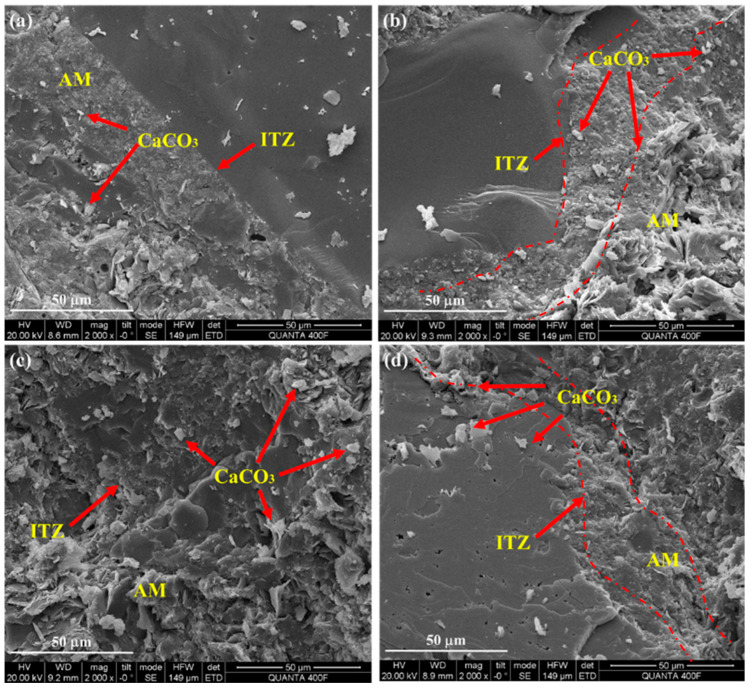
Microscopic SEM images of RA after carbonation modification under various gas pressures: (**a**) 0 MPa; (**b**) 0.01 MPa; (**c**) 0.5 MPa; (**d**) 1.0 MPa.

Figure 7 shows the microscopic SEM images of RA after carbonation modification under different gas pressures. The width and quantity of cracks reduced when the CaCO_3_ in the attached mortar significantly grew and filled the ITZ of RA when the gas pressure increased from 0 to 0.01 MPa. This was because the gas pressure promoted the diffusion rate of CO_2_ and speeded up the reaction rate of CO_2_ with hydration products. As the gas pressure rose to 0.5 MPa, the ITZ between the attached mortar and the original aggregate narrowed and became denser as carbonated products like CaCO_3_ gradually filled it. However, the ITZ between the attached mortar and the original aggregate was no longer visible as the gas pressure rose to 1.0 MPa, and flakes of CaCO_3_ started to show up in the mortar of RA that was attached. There was a limit to the further acceleration of the CO_2_ and hydration product reaction rate. The carbonation reaction rate cannot be accelerated by excessive pressure.

### 3.3. Effect of Moisture Content on the Physical Properties of RA

**Figure 8:**
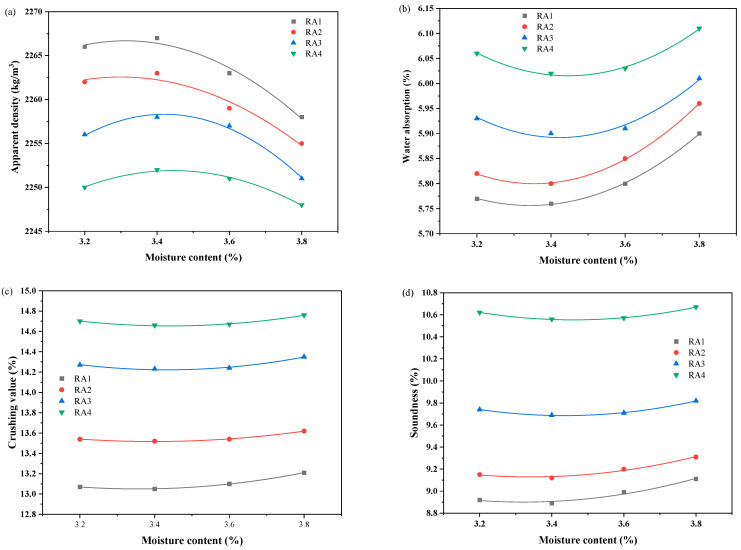
The physical properties of RA after carbonation modification with various moisture content: (**a**) apparent density; (**b**) water absorption; (**c**) crushing value; (**d**) soundness.

Figure 8 illustrates the effect of moisture content on the physical properties of RA. It was observed that the moisture content of RA had a significant influence on the carbonation modification of RA. When the moisture content of RA increased from 3.2% to 3.4%, the apparent density of RA increased, while the water absorption, crushing value, and soundness decreased. When the moisture content of RA further increased from 3.4% to 3.8%, the apparent density of RA decreased, and the water absorption, crushing value, and soundness increased. This is because when the moisture content of RA is low, the appropriate increase in the moisture content of RA is helpful to promote the dissolution of hydration products; when the moisture content of RA exceeds the optimal value, the diffusion rate of CO_2_ decreases, and the carbonation reaction rate also slows down [49,55]. It is shown that higher RA moisture content could not significantly improve the carbonation degree of RA. Wu et al. [56] revealed that this is because when the moisture content of the aggregates is high, water easily accumulates in the ITZ, especially in the area near the aggregates, which prevents the penetration of the CO_2_ and thus reduces the efficiency of the carbonation treatment. However, when the moisture content is too low, it cannot provide the amount of water required for the carbonation reaction. Therefore, the optimum RA moisture content range of RA carbonation modification is 3.2–3.4%.

**Figure 9:**
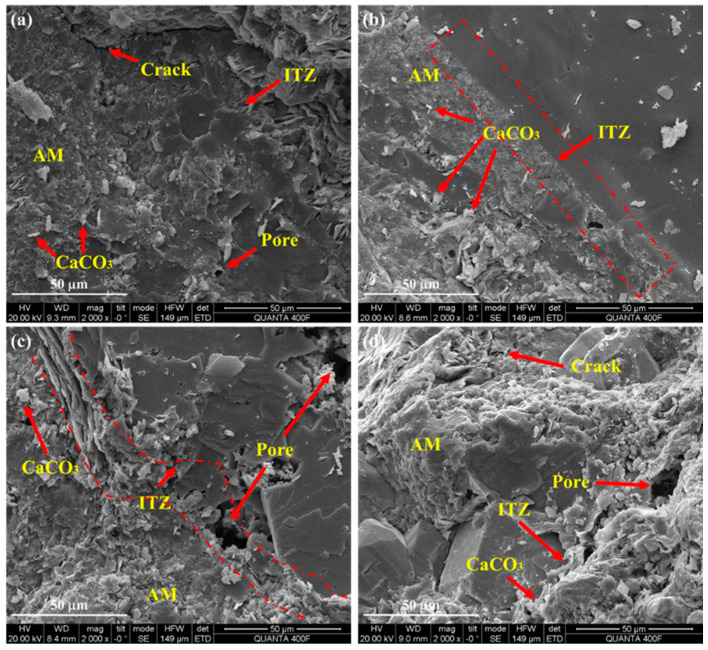
Microscopic SEM images of RA after carbonation modification with various moisture content: (**a**) 3.2%; (**b**) 3.4%; (**c**) 3.6%; (**d**) 3.8%.

Figure 9 shows the microscopic SEM images of RA after carbonation modification with different RA moisture content. The amount of CaCO_3_ in the attached mortar rose when the moisture content of carbonated modified RA increased from 3.2% to 3.4%, as seen in the microscopic SEM figure. As a result, ITZ gradually filled with carbonated materials and narrowed, the attached mortar densified, and the width and number of cracks decreased. The attached mortar gradually became loose and porous as the moisture content of carbonated modified RA rose from 3.4% to 3.8%. An excessively high moisture content in RA delayed CO_2_ diffusion, resulting in a decrease in the compactness of the RA’s internal structure [57].

## 4. Conclusions

This study focuses on the study of the effect of carbonation modification on the performance improvement of recycled coarse aggregates. It conducts carbonation modification experiments on RA to study the effect of carbonation modification factors including CO_2_ concentration, gas pressure, and moisture content on improving the characteristic parameters of low-quality RA in waste concrete, and the optimal effect of RA carbonation modification was analyzed. Finally, based on the results found on the improvement of apparent density, water absorption, crushing value, and the soundness of carbonated recycled coarse aggregate the main conclusions are as follows:

(a) With the increase in CO_2_ concentration, the apparent density of RA gradually increased, and the water absorption, crushing value, and soundness gradually decreased. When the CO_2_ concentration increases from 20% to 60%, CO_2_ can enter the interior of RA through pores and react with calcium ions, so high porosity will accelerate the carbonation reaction of RA. When the CO_2_ concentration is further increased to 80%, the cracks on the surface of AM are filled with carbonation products and become regular, which prevents further carbonation reaction to a certain extent;
(b) The carbonation reaction between the hydration products in AM and CO_2_ was accelerated when the gas pressure increased to 0.01 MPa; consequently, the physical properties of RA were enhanced significantly. The performance of RA was partially improved when the gas pressure exceeded 0.5 MPa; the CaCO_3_ flakes appeared in the attached mortar of RA, and the ITZ between the mortar and the original aggregate was no longer visible. Therefore, excessive gas pressure does not play a significant role in the degree of carbonation modification of RA;
(c) In the carbonation opposite evaluation, the effect of RA moisture content on the physical properties of RA is significant, and the optimal moisture content range of RA carbonation modification is 3.2–3.4%. However, the quality performance of RA decreased when the moisture content of RA increased from 3.4% to 3.8%. This is because when the moisture content of RA exceeds the optimal value, the diffusion rate of CO_2_ decreases, and the carbonation reaction rate decreases, accordingly;
(d) In general, it is recommended to maintain a CO_2_ concentration between 40% and 60% during the carbonation modification process. The carbonation effectiveness increases with pressure, and from an economic perspective, a carbonation pressure of 0.01 MPa is suggested. The moisture content of the recycled aggregates is ideally within the range of 3.2–3.4%; excessively high moisture content can hinder CO_2_ diffusion and reduce carbonation efficiency.

## Figures and Tables

**Table 1:** Physical properties of RA.

Type	Apparent Density kg/m^3^	Water Absorption %	Crushing Value %	Soundness %
RA1	2231	6.38	14.36	9.86
RA2	2226	6.44	14.91	10.15
RA3	2219	6.57	15.74	10.82
RA4	2212	6.72	16.24	11.81

**Table 2:** Carbonation condition.

Condition	CO_2_ Concentration (%)	Gas Pressure (MPa)	Moisture Content (%)
1	20, 40, 60, 80	0.01	3.4
2	20 ± 3	0, 0.01, 0.5, 1.0	3.4
3	20 ± 3	0.01	3.2, 3.4, 3.6, 3.8

## Data Availability

The original contributions presented in this study are included in the article. Further inquiries can be directed to the corresponding author.
